# Mapping Ethics Education in Accounting Research: A Bibliometric Analysis

**DOI:** 10.1007/s10551-021-04846-9

**Published:** 2021-06-10

**Authors:** Tamara Poje, Maja Zaman Groff

**Affiliations:** School of Economics and Business, University of Ljubljana, Kardeljeva pl. 17, 1000 Ljubljana, Slovenia

**Keywords:** Accounting ethics education, Citation analysis, Bibliographic coupling, Historiography, Co-word analysis, I23, M41

## Abstract

The attention being paid to ethics education in accounting has been increasing, especially after the corporate accounting scandals at the turn of the century. This paper provides a comprehensive overview of the existing research in the field of ethics education in accounting. To synthesize past research, a bibliometric analysis that references 134 primary studies is performed and three bibliometric methods are applied. First, we visualize the historical evolution of ethics education in accounting research through historiography. Second, we use bibliographic coupling to identify clusters of ethics education in accounting research before, during, and after major corporate scandals. Third, we perform a co-word analysis to connect the identified patterns into a map of a contextual space. The results reveal, in each decade, not only an increasing academic focus on this field of research, but also an increasing number of different research clusters. While the clusters Factors affecting moral judgement, Perception of ethics, and Lack of ethics topics in the last research period develop further from the respective clusters in the previous periods, Accounting beyond technical skills, Integration of ethics in accounting education, Use of developed ethics frameworks, and Professional values on the contrary develop anew in the last decade, as a consequence of a growing demand for teaching ethics. Overall, the paper presents the development patterns of ethics education in accounting research and sets up a research agenda that encourages future research.

## Introduction

The importance of ethics in accounting has vastly increased following the corporate scandals at the turn of the century. These scandals reflect a serious lack of ethics in both the field of financial reporting, which is primarily intended to provide true and fair representation to external users of financial statements, and accountability to the general public. Low et al. (2008) point out that corporate scandals have always drawn attention to the role of accountants who simultaneously assisted in financial management, preparation, and auditing of financial statements, and that “*recent corporate scandals have set a new low for the accounting profession*” (Low et al., 2008, p. 222).

To prevent further corporate scandals, different legislative changes, including the Sarbanes–Oxley Act, Directive 2006/43/EC, Directive 2014/56/EU and Regulation 537/2014, were adopted. In regaining public trust, professional codes proved to be another essential part. Accordingly, the Code of Ethics for Professional Accountants issued by the International Ethics Standards Board for Accountants (IESBA), was revised.

To change behavior and mindset, setting up policies is not enough ─ moral development is needed as well. Enhancement of ethics education is therefore a viable solution in addressing the ethical crisis in the accounting profession (Jackling et al., 2007) that has certainly encouraged research in ethics education itself (De Lange et al., 2006; Dellaportas, 2006; McPhail, 2001; Melé, 2005; Roxas & Stoneback, 2004).

The main objective of the paper is to provide a comprehensive overview of the existing research in the field of ethics education in accounting, while at the same time improving its understanding and driving its future research. More specifically, our goals are to firstly, outline influential papers that have shaped or reformed this field and provide an overview of the historical developments in the field of research, secondly, examine the developments, and thirdly, develop a research agenda that fosters future research.

Most studies of ethics education in accounting present a static view of the current situation. However, since the topic is evolving dynamically over time, the present study instead provides a dynamic perspective on how this field of research has advanced over the last three decades. The resulting development patterns of ethics education in the accounting research, combined with the current developments in the business environment, provide a basis for not only predicting future developments in this field, but also encouraging future research. To the best of our knowledge, this is the very first comprehensive literature review based on a bibliometric analysis in the field of ethics education in the accounting research. As such, it aims to examine the proposed research area in a transparent and quantitative way. We believe that the bibliometric methods applied overcome to some extent the problems of the subjectivity that is associated with peer review and result in more objective outcomes.

Three bibliographic methods are used in the research to achieve the objectives set. Firstly, historiography is performed to show the development of theoretical approaches. Secondly, bibliographic coupling is used to identify and analyze the individual clusters of the academic literature and the relations between them in the periods before, during, and after the corporate accounting scandals. And lastly, a co-word analysis is conducted to show the thematic landscape.

Based on the bibliometric analysis, we look for a developmental path in the ethics education in accounting research. The results reveal that research interest has been increasing throughout the observed periods with a relatively slow increase during the corporate scandals, but a considerable increase of research interest in the period following the scandals, when the number of published articles quadrupled and the number of different clusters doubled, when compared to the previous period. For the last research period, seven research clusters that evolved within the ethics education in accounting research are identified. Namely, while the clusters *Factors affecting moral judgement, Perception of ethics*, and *Lack of ethics topics* develop further from the respective clusters in the previous periods, *Accounting beyond technical skills, Integration of ethics in accounting education, Use of developed ethics frameworks*, and *Professional values* on the contrary develop anew in the last decade, as a consequence of a growing demand for teaching ethics. In the last research period, the research questions, such as why ethics education is needed, are replaced by the questions related to the contents of ethics courses and the different approaches to teaching ethics (e.g. stand-alone course or integrated across the curriculum, the use of innovative teaching methods). Among the factors influencing the ethical decision-making process, both individual and situational factors are explored, with the emphasis on the latter (e.g. predispositions, pressure, culture).

The research findings primarily relate to educators and researchers in the field, where the former are provided with relevant sources that they can use to design courses, while the latter obtain a comprehensive review of the most relevant research that has already been conducted in the field, together with the current trends and possible avenues for further research.

The paper is structured as follows. After the introduction, the background section first indicates the role of the corporate accounting scandals in the enhancement of the ethics education in accounting, then outlines the prominence of the ethics education in the accounting research, and in the end presents the arguments for the selected bibliometric methods. In the following section, the methodology used to perform the analysis of ethics education in the accounting literature is presented. Next, we identify the thematic landscapes and theoretical approaches, define clusters and contextualize the main findings for all periods. The study concludes with the discussion, viable areas for future research and conclusion sections that include research limitations.

## Background

The accounting profession consists of financial accountants, management accountants, auditors, tax consultants, valuation specialists, financial analysts and other professionals. What they all have in common, aside from accounting knowledge, is their daily involvement in making ethical choices. Questionable accounting practices, including ethical concerns, have already existed long before the most famous corporate accounting scandal Enron (Duska et al., 2018). Some noticeable early examples cover Yale Express System (1965), Equity Funding (1973), and Waste Management Scandal (1998). Although Enron and WorldCom are most known of all, the scandals occurred all over the world (see Table 1).

**Table 1:** Some of the most known corporate scandals by region

Region	Some of the most known scandals
North America	Enron (2001), Global Crossing (2002), WorldCom, Tyco (2002), HealthSouth (2003), Freddie Mac (2003), American International Group (2005), Lehman Brothers (2008), and Satyam scandal (2009)
Europe	Parmalat (2003), Olympus (2011), Swiss Leak (2015), Panama Papers (2016), CumEx-Files (2017), and Danske Bank (2018)
Asia	SK Global (2001), Olympus (2011), Toshiba (2015), and Luckin Coffee (2020)
South America	Petrobras (2014) and Deloitte Brazil (2016)
Australia	Harris Scarfe (2000), OneTel (2001), and HIH Insurance (2001)
Africa	Zuma (2016)

Aside from the consequences directly related to the stakeholders, the effects were more far-reaching, and revealed themselves in the reduced quality of financial reporting (Ball, 2009) and in investors losing confidence in financial information, resulting in a severe loss of market capitalization (Petra & Spieler, 2020). In addition, this was followed by a lack of public trust which is “essential not only for preserving respectability but also for ensuring the survival of accounting’s status as a profession” (Carnegie & Napier, 2010, p. 360). Among the many reasons which contributed to the corporate accounting scandals, lack of auditors’ independence (Sikka, 2009; Unerman & O’Dwyer, 2004) and professional skepticism were outlined (Benston & Hartgraves, 2002). The independence in the accounting profession is at the core of the International Code of Ethics for Professional Accountants. To mitigate such scandals in the future, ethics education proves just as important, since it improves professional skepticism (Ratna & Anisykurlillah, 2020).

Studying not only the efficiency of different approaches to teaching ethics in accounting programs, but also the effects of ethics education on the moral development of accounting students, the importance of developing values and virtues in accounting students becomes therefore relevant and timely. Accounting ethics education is outlined by Uysal (2010) in a comprehensive bibliometric analysis of the business ethics research with an accounting focus as one of the three areas that have, along with the Moral cognitive development and Implications for ethical decision-making models, evolved in the accounting business ethics literature. Moreover, in the review by Bampton and Cowton (2013), education is outlined as a subfield within the accounting ethics literature.

Due to an increasing number of relevant papers, identifying the most important contributions in the field by studying the literature is time-consuming and subjective. To overcome these difficulties and to objectify the results at least partly, a bibliographic analysis was developed, consisting of various bibliometric methods, among which citation analysis is most frequently used. The idea behind citation analysis is that the most frequently-cited authors and papers are also most relevant to scholars and researchers (Garfield, 1979). Zupic and Čater (2015) differentiate between five main bibliographic methods, namely the citation analysis, co-citation analysis, bibliographic coupling, co-author analysis and co-word analysis. The methods differ based on the unit of each analysis, or in other words, it is common for the first three methods that they use citation data as an input, while the input in the co-author analysis comes from authorship data and in the co-word analysis from words.

The bibliographic analysis in the field of accounting started with studying the flow of information within accounting journals (McRae, 1974), the influence of a particular journal (Brown & Gardner, 1985), and ranking accounting journals by their influence (Tahai & Rigsby, 1998). All in all, bibliographic analysis remains to this day a prominent research method in accounting (Ameen & Guffey, 2017; Bisman, 2011; Chan et al., 2009; Ezenwoke et al., 2019; Guffey & Harp, 2017; Uysal, 2010). Citation analysis conducted by Ameen and Guffey (2017) in the field of teaching and curriculum innovation in accounting, which reveals that out of the six most cited articles three are related to the topic of ethics education, shows how important this field of research actually is.

## Methods

### Analyses

To identify the research areas developed in the field of ethics education in accounting, we use the clustering method as defined in the bibliometric literature (van Eck & Waltman, 2017; Waltman et al., 2010). The advantage of this method is the reduction of subjectivity, since qualitative data are analyzed quantitatively. However, the interpretation of quantitative results remains subject to subjectivity. Further, to show the structure of the research area and the development path, science mapping is used (Zupic & Čater, 2015).

#### Historiography

Bibliographic coupling shows a static picture of a research field. Since we are also interested in the field’s development over time, we perform historiography in CitNetExplorer. Historiography analyzes the chronological development of the research field by visualizing the most important publications and showing how articles build on each other. The tool enables the identification of the most important publications in the field in chronological order and reveals citations between them. In addition, it uses citation networks of individual publications as the data for analysis (van Eck & Waltman, 2014).

#### Co-word Analysis

We perform a co-word or co-occurrence analysis, representing a content analysis that connects words or noun phrases in the title or abstract. Based on the connections, a conceptual structure of the topic can be built, in other words, the more times the terms appear together, the stronger the connection of the concepts (He, 1991). Among the existing bibliographic methods, co-word analysis is the only one that uses text data instead of bibliographic data as a source for the analysis (van Eck & Waltman, 2019) to show the thematic landscape. It analyses the documents’ content, while other methods are more focused on searching for connections only through the citation analysis. The major idea of the co-word analysis is to connect any identified patterns into a map of contextual space. And it is the sequence of such maps for different periods that shows the conceptual transformation (Coulter et al., 1998).

#### Bibliographic Coupling

We use bibliographic coupling as a cross-citation technique which refers to two documents that have at least one common reference. This method searches for bibliography overlaps and since it looks for citing publications, it is a retrospective (static) similarity measure, not depending on a certain point in time. The method is in addition used to determine recent contributions and research trends (Vogel & Güttel, 2013). And although bibliometric methods are generally useful, they are nevertheless not self-sufficient, as they do not provide any information about the content of the analyzed papers. Within our research, the bibliographic coupling method is used primarily to map and analyze research in the field of ethics education in accounting.

### Sampling and Data

Our analysis is based on the Web of Science data source. Despite all the existing articles from the field of ethics education in accounting not being included in the Web of Science, we use this database for the following reasons. Firstly, the Web of Science covers the widest time span and comprises articles that are over 40 years older than the articles in other databases. As an example, the Web of Science includes articles from 1956, while Scopus from 1996 (Meho & Yang, 2007). In any case, older articles are necessary for the reason of performing a historical comparison. Secondly, the database also covers the Social Science Citation Index that, due to an independent and thorough editorial process, in the end ensures the overall journal quality. Thirdly, the Web of Science was designed particularly for citation analysis (Falagas et al., 2008).

To derive the data set of the studies for the analysis, we first used keywords *accounting*, *education*, and ethic*^1^ as a search engine in the Web of Science. In total, 385 articles matching all three keywords were found in July 2020. Next, we limited the search to the following science categories: business finance, business, education educational research and ethics, which scaled the list of relevant articles down to 273. Third, we defined article as a document type (after which 205 articles remained) and English as a document language (after which 192 articles remained). To prevent any omission of the relevant articles, we also ran searches with different combinations of keywords, including *teaching*, *accountant* and similar, all resulting in comparable sets of articles.

For the set of the remaining 192 articles, we downloaded and read all the abstracts to examine, if the content of the articles is in line with our research topic. We excluded those articles that were not directly related to our field of research. The majority of the excluded articles were closely related to two keywords, while the third one was only mentioned. The mere mention of the keyword was sufficient for an inclusion by the program, however, the manual check did not show a match with the research topic. Based on our judgment, additional 58 articles were excluded as non-relevant for the analysis, resulting in the final selection of 134 primary articles to be included in further research. Only one article was dated before 1991 (1939) and was for that reason eliminated from further analysis.

**Fig. 1:**
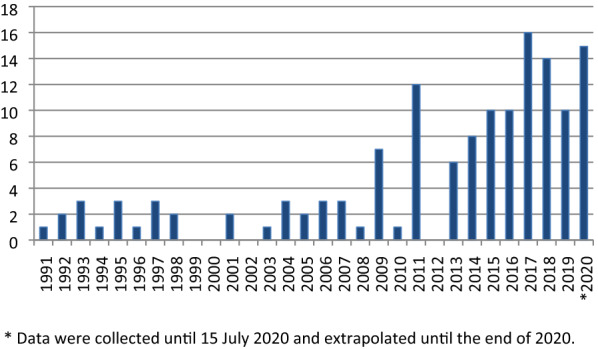
Number of articles published by year.

Figure 1 presents the number of articles in the final sample published by year. The vast majority of articles were published in the Journal of Business Ethics, as presented in Table 2.

**Table 2:** Top 5 journals based on the number of published articles

Journal	Number of articles
Journal of Business Ethics	58
Advances in Accounting Education: Teaching and Curriculum Innovations	8
Accounting Education	7
Critical Perspectives on Accounting	5
Research on Professional Responsibility and Ethics in Accounting	4

The data were analyzed with the VOSviewer software. For the analysis purpose, we divided the articles into three time periods, namely decades: 1991–2000, 2001–2010 and 2011–2020. The first decade corresponds to the period before the major corporate scandals, the second decade coincides with the scandals, while the third decade represents the period after the scandals. The selection of time periods enables an identification of the possible impact of corporate accounting scandals and the related public mistrust in the accounting profession on the ethics education in accounting research. Table 3 presents the five most cited articles for each period.

**Table 3:** The list of 5 most cited articles for each period

1991–2000		2001–2010		2011–2020	
Citations	Article	Citations	Article	Citations	Article
89	Eynon et al. (1997)	150	Cohen et al. (2001)	31	Chabrak and Craig (2013)
65	Green and Weber (1997)	116	Roxas and Stoneback (2004)	22	Martinov-Bennie and Mladenovic (2015)
48	Jones and Hiltebeitel (1995)	90	De Lange et al. (2006)	19	O’Leary and Stewart (2013)
45	Karcher (1996)	86	Dellaportas (2006)	18	Musbah et al. (2016)
44	Fischer and Rosenzweig (1995)	84	McPhail (2001)	17	Tweedie et al. (2013)

To analyze the bibliographic data, we used BibExcel that shows co-occurrences of references in the articles’ bibliographies (Persson et al., 2009). BibExcel was applied to analyze secondary documents (citation within citation) which are documents cited by primary articles (cited articles). The five most cited documents among secondary documents are Rest (1986; cited in 33 primary papers), Loeb (1988; cited in 24 primary papers), Dellaportas (2006; cited in 21 primary papers), Jones (1991; cited in 19 primary papers), and Blanthorne et al. (2007; cited in 18 primary papers). In the most cited paper, Rest (1986) develops the *Defining Issue Test* (DIT), which is the most widely used instrument for measuring an individual’s level of moral development, where the individual identifies the ethical issues for each ethical dilemma.

**Fig. 2:**
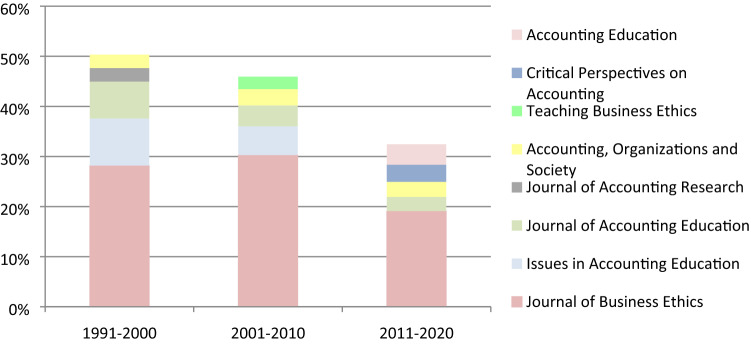
The list of the 5 most cited journals among secondary documents for each period and their coverage *among all citations*

In all three analyzed periods, most of the cited articles were published in the Journal of Business Ethics. However, in the last period, the percentage of the cited papers published in the Journal of Business Ethics dropped due to the increased number of different cited sources (1991–2000: 57, 2001–2010: 185, 2011–2020: 436). Since researchers in the field of ethics education in accounting apply knowledge also from other fields, such as medicine, nursing, physiology, sociology, business, innovation, law, etc., the percentage of the cited articles per journal is reduced, when compared to the previous two periods (Fig. 2).

## Results

### Historiography

**Fig. 3:**
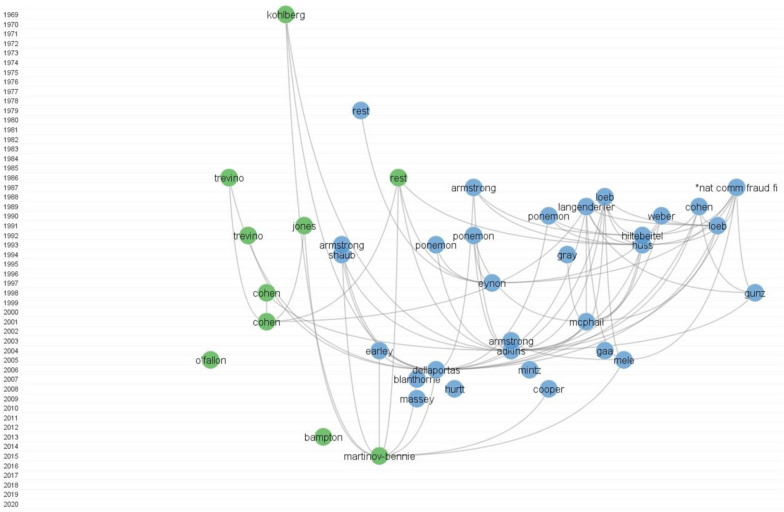
Citation network of the evolution of ethics education in accounting research. *Note*: *National Commission on Fraudulent Financial Reporting. To increase visibility, only the most important articles are shown

To understand the development of the ethics education in accounting research we perform historiography on all the main documents. Historiography was conducted in CitNetExplorer (van Eck & Waltman, 2014) on a full sample of primary papers, as described earlier. Since analyzing a large number of citation relations may result in unclear results, the full citation network was reduced in CitNetExplorer in two ways. First, by defining the minimum number of citation relations required for a publication to be included in the analysis, we followed the procedure of van Eck and Waltman (2014), where only publications with at least ten internal citation relations were included in the analysis. Second, by applying the transitive reduction method, where the program distinguishes between essential and non-essential citation relations in the network, only the essential relations (no other relations connect two publications) are shown in the final result. For additional explanation on the transitive reduction method see van Eck and Waltman (2014). Figure 3 shows two main research streams (cluster 1, green; cluster 2, blue) that have been established, using CitNetExplorer, where in the last five years, cluster 1 (green) has become the dominant one.

The first cluster consists of 41 publications (Fig. 3, green), starting with a seminal work by Kohlberg (1969), who developed the theoretical foundation of moral development. Based on the latter, the theory then further developed with the works of Rest (1986) and Trevino (1986). Rest (1986) developed a four-step model of moral development, while Trevino (1986) built a competitive model, adding additional individual and situational variables into the model. Trevino’s and Rest’s models both outline cognitive moral development as crucial for judgment. A few years later, Jones (1991) developed a new model based on Rest’s theory, introducing moral intensity as a factor affecting the four stages.

The first themes within this cluster are oriented towards theory development and are followed by the development of more practice-oriented themes, addressing the importance of real-world dilemmas related to profession (Loeb, 1988; McPhail, 2001). Recent articles continue to add to the development of a theoretical foundation by connecting the existing topics (Martinov-Bennie & Mladenovic, 2015), providing literature reviews (Bampton & Cowton, 2013; O’Fallon & Butterfield, 2005) or pointing out the current stand on the topic (Marques & Azevedo-Pereira, 2009).

The second cluster (Fig. 3, blue) is the largest and consists of 96 publications. The second research stream starts with Rest’s (1979) development of a measurement instrument, the *Defining Issue Test (DIT),* which is a self-report measure that gives quantitative values to moral issues. Therefore, the focus of the majority of the research in the second cluster is on the research using DIT, or a combination of DIT and other measurement instruments (Armstrong, 1987; Dellaportas, 2006; Eynon et al., 1997, etc.).

One of the themes that emerges in the 1990s within the second cluster is the *factors influencing moral judgment.* Authors investigate the effect on moral judgment of individual factors, such as gender (Adkins & Radtke, 2004; Jones & Hiltebeitel, 1995) and age (Adkins & Radtke, 2004; Jones & Hiltebeitel, 1995), and situational factors, such as social pressure (Mayhew & Murphy, 2009; O’Leary et al., 2007) and education (Cooper et al., 2008; Halbesleben et al., 2005; Hiltebeitel & Jones, 1992; McNair & Milam, 1993; Mohd Ghazali, 2015). After 2000, research on ethics education in accounting spread to non-Western countries, including Turkey (Karaibrahimoğlu et al., 2009), Malaysia (Marzuki et al., 2017; Mohd Ghazali, 2015), China (Driskill & Rankin, 2020; Liu, 2018) and Tunisia (Arfaoui et al., 2016). In the 2010s, research became more detailed and focused on different topics, such as the benefits of teaching ethics (Arfaoui et al., 2016), questions related to course development (Kidwell et al., 2011; Sorensen et al., 2017; Tweedie et al., 2013), and extending the accounting knowledge beyond technical skills (Gordon, 2011).

### Bibliographic Analysis

Next, the data collected and selected were analyzed using two bibliographic methods, i.e. co-word analysis and bibliographic coupling. We performed both methods for each period, with the clustering solution depending on the minimum occurrences or citations defined, resolution parameter and minimum cluster size. As recommended by van Eck and Waltman (2017), we performed different clustering solutions in order to achieve good explanatory power. Consequently, there are methodological differences between the periods. Since the aim of our co-word analysis was to primarily identify approximately 25 most frequently used words, we adjusted the minimum occurrence in each period accordingly. The resolution parameter and minimum cluster size were both set to the default value (1), except for the second period, for which the resolution parameter was set at 0.9. As for bibliographic coupling, due to the manageable number of publications in the field, the minimum citations defined in bibliographic coupling remained as the default value (0), as well as the minimum cluster size (1), in all three periods. To improve explanatory power, the resolution parameter was adjusted to 0.8 in the first period, while being set to the default value (1) in the last two periods.

The bibliographic analysis based on co-word analysis and bibliographic coupling was performed separately for each of the three research periods. For every period, we define the research fields obtained by each of the two methodological approaches. As the co-word analysis is based on article content (words and phrases), it results in thematic landscapes or clusters representing the terms that are used together most frequently. Consequently, it is already the words or phrases within each cluster that define the cluster’s content and there is thus no need to label the clusters based on the supplementary content analysis. On the other hand, bibliographic coupling provides clusters of articles that are grouped together based on their references, not their content. Therefore, for each cluster obtained by bibliographic coupling, we perform an additional manual qualitative analysis of its articles’ content to assign pertinent cluster labels (names). Although the advantage of the bibliographic analysis is reduction of subjectivity, cluster naming nevertheless remains subject to subjectivity.

**Fig. 4:**
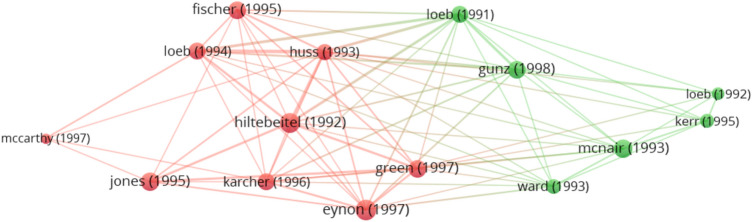
Period 1991–2000 with clusters for ethics education in accounting based on bibliographic coupling *Note*: a) colors represent the clusters, b) lines represent the connections between the items, c) the size of both the label and the circle represents weight of an item, and d) the distance between the items and the weight of the lines represents the relatedness between the items

**Fig. 5:**
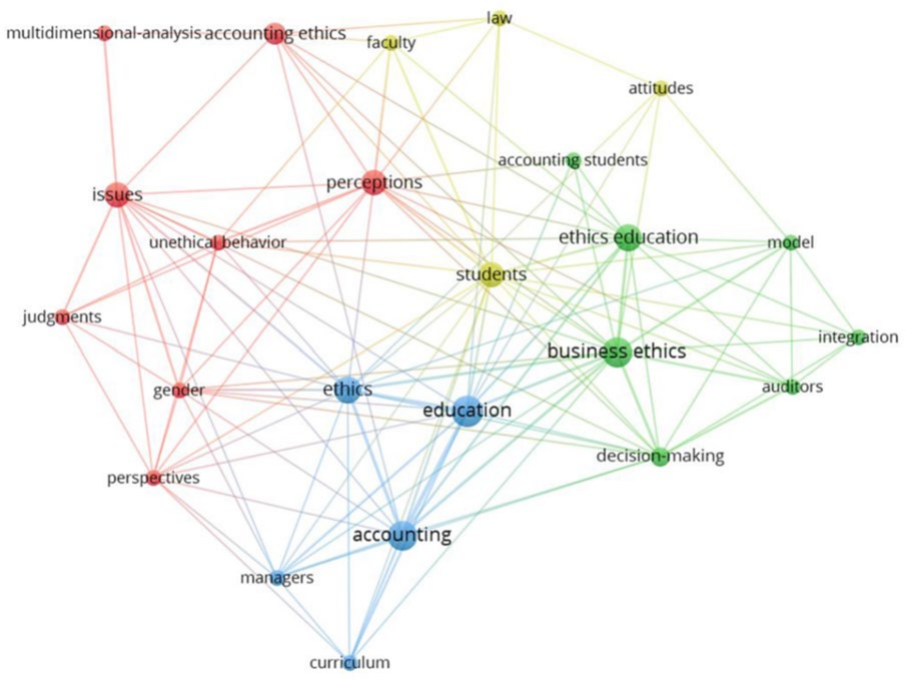
Co-word analysis for the period 2001–2010 *Note*: a) colors represent the clusters, b) lines represent the connections between the selected words, c) the size of both the label and the circle represents the weight of a word, d) the distance between words and the weight of the lines represents the relatedness between the words

**Fig. 6:**
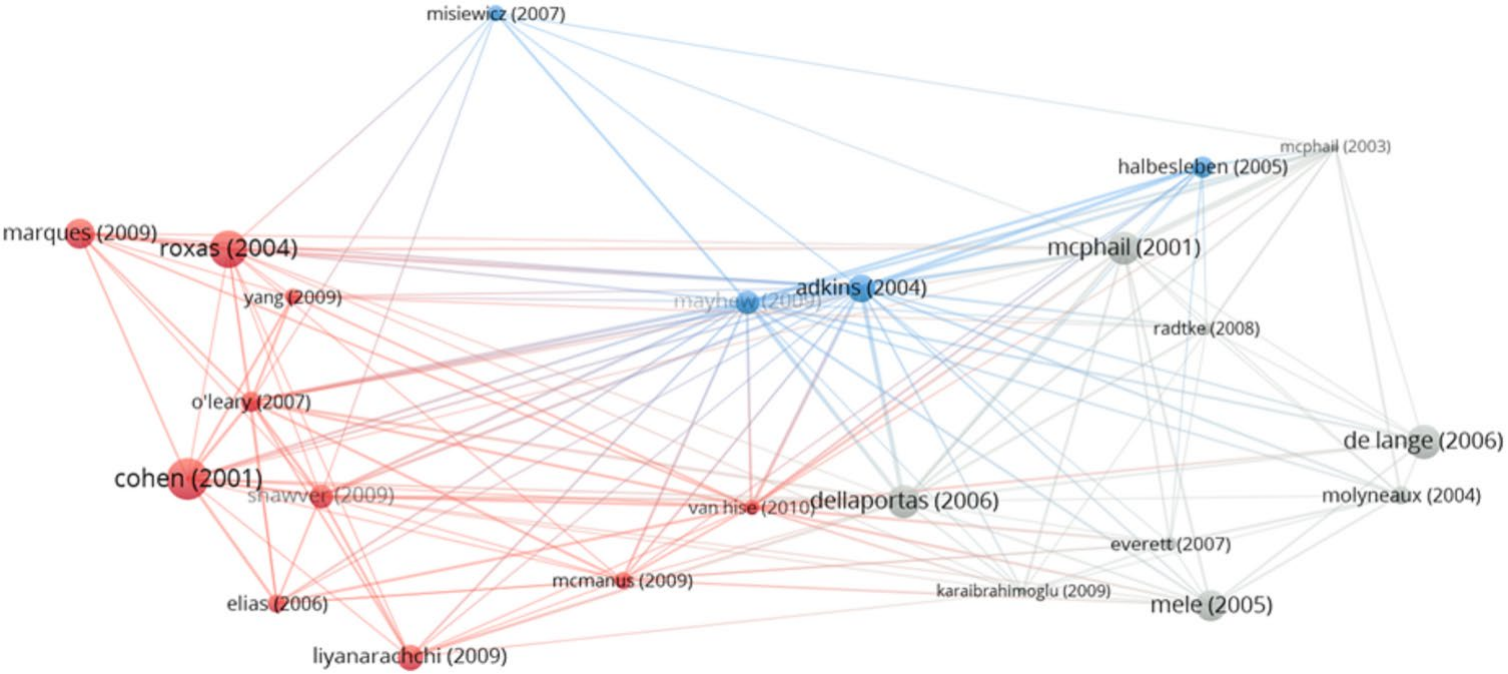
Period 2001–2010 clusters for ethics education in accounting based on bibliographic coupling *Note*: a) colors represent the clusters, b) lines represent the connections between the items, c) the size of both the label and the circle represents weight of an item, and d) the distance between the items and the weight of the lines represents the relatedness between the items

**Fig. 7:**
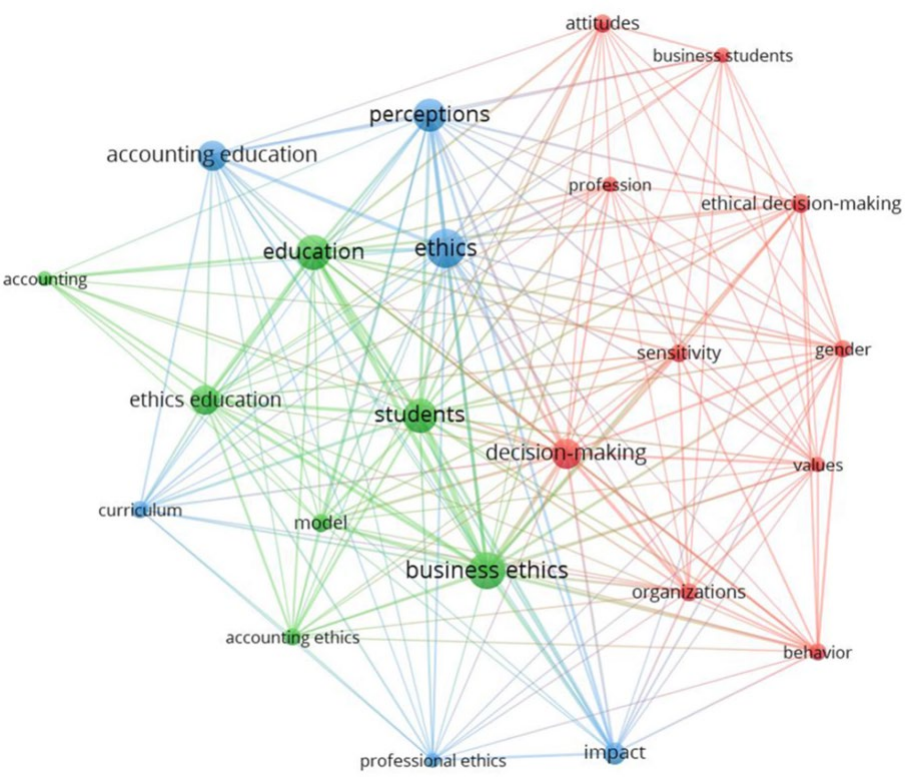
Co-word analysis for the period 2011–2020 *Note*: a) colors represent the clusters, b) lines represent the connections between the words, c) the size of both the label and the circle represents the weight of a word, d) the distance between the words and the weight of the lines represents the relatedness between the words

**Fig. 8:**
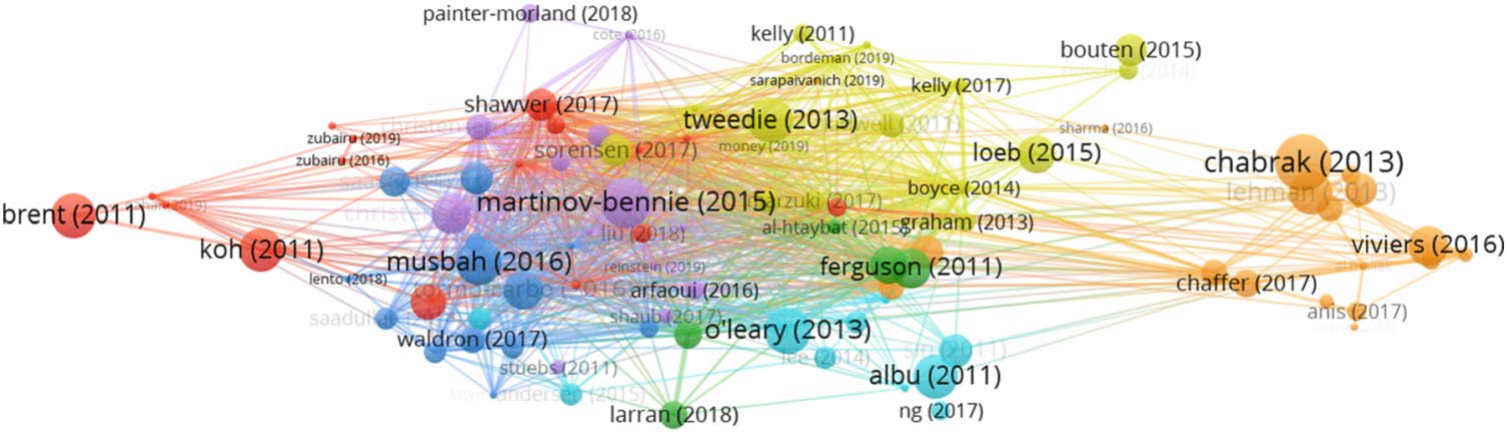
Period 2011–2020 clusters for ethics education in accounting based on bibliographic coupling. *Note*: a) colors represent the clusters, b) lines represent the connections between the items, c) the size of both the label and the circle represents weight of an item, and d) the distance between the items and the weight of the lines represents the relatedness between the items

For each period, we exhibit the clusters identified with VOSviewer for both methodological approaches, where each item is assigned to exactly one cluster based on the method used (bibliographic coupling or co-word analysis). For presentation purposes, the clusters in Figs. 4, 5, 6, 7, and 8 are represented by different colors. The weight of an item determines the size of the label and the circle. Weight is defined by total link strength, which is the cumulative strength of the links of an item with other items. Two publications have greater coupling strength, the more citations to other publications they share. The lines between the publications represent the links between them (van Eck & Waltman, 2019). Moreover, Tables 4, 5 and 6 provide a list of clusters in each research period. In addition to the cluster number and color, the cluster label (name) based on the manual content analysis is provided along with a list of the five most cited references. This section presents the main findings for each period, followed by a discussion describing the patterns of development.

**Table 4:** Cluster labels and articles in clusters for ethics education in accounting in the period 1991–2000

Cluster number	Color (Fig. 4)	Cluster label	Number of documents	Five most cited references
1	Red	Factors affecting ethical decision-making process	9	Eynon et al. (1997), Fischer and Rosenzweig (1995) Green and Weber, (1997), Jones and Hiltebeitel (1995), Karcher (1996)
2	Green	Lack of ethics topics	6	Gunz and McCutcheon (1998), Kerr and Smith (1995), Loeb (1991), McNair and Milam (1993), Ward et al. (1993)

#### Research Fields for the Period 1991–2000

For the 16 articles published in the period 1991–2000, the co-word analysis identifies only one cluster consisting of the following words: *business, education, future managers, moral development*, and *society course*. The list of most frequently used words reveals that research flow builds on the importance of teaching ethics, as a result of its effect on the accounting profession. Identification of a single cluster may occur, due to the limited number of articles published in this period.

For bibliographic coupling, the largest set of the connected items consists of 15 items (Table 4), with the total number of clusters identified standing at 2 (Fig. 4), all articles published in the Journal of Business Ethics.

Even before the outbreak of the major corporate accounting scandals, researchers were aware of the inadequate treatment of ethical issues in the classroom. As reported in the research published between 1991 and 2000, ethics education was at the time already included in some parts of the accounting curriculum, but not sufficiently (Gunz & McCutcheon, 1998; Kerr & Smith, 1995; Loeb, 1991; Loeb & Rockness, 1992; McNair & Milam, 1993). We label this research cluster *Lack of ethics topics*. McNair and Milam (1993) reported that although ethics education is included in accounting courses, the scope is nevertheless limited and needs to be expanded. It was already then believed that ethics should be integrated into the accounting curriculum in a way that enhances students’ moral development which can be achieved by integrating ethics content into the core accounting courses rather than having separate ethics courses (Loeb & Rockness, 1992; McNair & Milam, 1993).

Research in this period reveals that one of the reasons for the lack of ethics topics may be found in the insufficient academic commitment. Despite an increase in the accounting ethics articles, little research has been done on the topic (Gunz & McCutcheon, 1998). The professors who participated in McNair and Milam’s (1993) study agree that an increased coverage of ethics topics in accounting courses is much needed. Further, they pinpoint the lack of time and available materials as the main causes of the problems associated with ethics education in accounting. A lack of ethics can have a negative impact on both the accounting profession and society, however, although ethical issues are not adequately addressed in accounting courses, the research identifies positive trends toward teaching ethics in accounting (Loeb, 1991).

One of the biggest challenges facing accounting educators is how to integrate ethics topics into the curriculum (Huss & Patterson, 1993; Loeb, 1994; McCarthy, 1997), as there is still no consensus on the effect of ethics courses on ethical orientation. While research by Hiltebeitel and Jones (1992) shows that ethics integration does in fact affect ethical orientation, McCarthy (1997), on the contrary, finds no differences. Moreover, it is further ascertained that implementation of ethics education alone is not enough, since it should also be defined why and how to integrate it into the curriculum (Loeb, 1994). In addition, ethics should be taught in a way that encourages students’ critical thinking about ethical dilemmas, while the purpose of integrating ethics into the curriculum should be to improve students’ moral development (Fischer & Rosenzweig, 1995).

Within the cluster labeled *Factors affecting ethical decision-making process*, authors also study the effects of personal characteristics (Hiltebeitel & Jones, 1992), in addition to ethics education. While Eynon et al. (1997) report that certified accountants have lower levels of moral judgment, when compared to other professions, that is lower than the average adult and average student, Green and Weber (1997) on the contrary find no differences in moral judgment, when comparing junior accounting and other business students. Therefore, no consensus exists to date between researchers as to whether more or less ethical students choose accounting as their major. On the other hand, differences are reported when comparing senior-level accounting and other business students, with the latter presenting lower levels of moral judgment (Green & Weber, 1997). It is necessary to add here that senior accounting students in the sample were exposed to a professional code of ethics, which may have affected their moral judgment. Jones and Hiltebeitel (1995) find that personal characteristics (age, gender, education), organizational expectations, and internalized expectations do in fact affect moral judgment. The overall research also shows that the moral development of accountants is an ongoing process, and that ethics training should continue after formal education. And since accountants with lower levels of moral judgment are less supportive of ethical education, Eynon et al. (1997) suggest mandatory ethical training.

It is obvious that the importance of ethics education in accounting already drew the attention of researchers in the period 1991–2000 before the major corporate accounting scandals. However, during the next period (2001–2010), the number of articles in this field of research increased by 44%.

#### Research Fields for the Period 2001–2010

Corporate scandals that occurred in the period 2001–2010 encouraged many researchers to pay additional attention to ethics education in accounting:

“*Such scandals have again questioned the business and accounting practices of these firms and the role played by their auditors*.” (Dellaportas, 2006, p. 391)

“*The scandals* […] *remind us that accounting programs still need to teach ethical conduct*.” (Shawver & Sennetti, 2009, p. 663)

“*Thus, considerable steps have been made in ethical accounting education, but, after the well-known recent accounting scandals, it seems absolutely essential to pay increasing attention to ethics in accounting and to improve ethical education for accountants*.” (Melé, 2005, p. 97)

“*In light of the myriad accounting and corporate ethics scandals of the early 21st century, many corporate leaders and management scholars believe that ethics education is an essential component in business school education*.” (Halbesleben et al., 2005, p. 385)

Based on a selection of 23 articles for the period 2001–2010, the co-word analysis shows a more precise picture of the thematic landscape than it does in the previous period. To narrow down the results, the minimum number of occurrences of keywords was defined as 2. Out of 143 keywords, 24 met the reduction criteria.

A notable cluster derived by the co-word analysis (Fig. 5, red) consists of words *judgments*, *perceptions*, *unethical behavior*, *gender*, *perspectives*, *issues*, *multidimensional analyses* and *accounting ethics*. On one hand, this cluster reveals a continued interest of researchers in the factors affecting ethical decision making, but on the other hand, it suggests an increasing importance of the perception of ethics. Perception is further connected with two main themes, one related to students (yellow) and the other related to professionals (blue). The last cluster (green) relates to the notion that the success of teaching ethics depends on the way ethics content is delivered. Despite the fact that the manner of teaching ethics is at this point in time not yet identified as an individual research cluster, the co-word analysis reveals that many authors already started exploring this field.

Based on bibliographic coupling, the largest set of connected items consists of all articles (Table 5). The total number of the clusters identified is three (Fig. 6), of which the majority (19) were published in the Journal of Business Ethics.

**Table 5:** Cluster labels and articles in clusters for ethics education in accounting in the period 2001–2010

Cluster number	Color (Fig. 6)	Cluster label	Number of documents	Five most cited references
1	Red	Factors affecting ethical decision-making process	10	Cohen et al. (2001), Liyanarachchi and Newdick (2009), Marques and Azevedo-Pereira (2009), Roxas and Stoneback (2004), Shawver and Sennetti (2009)
2	Grey	The need to teach ethics	9	De Lange et al. (2006), Dellaportas (2006), McPhail (2001); Melé, (2005), Molyneaux (2004)
3	Blue	Perception of ethics	4	Adkins and Radtke (2004), Halbesleben et al. (2005), Mayhew and Murphy (2009), Misiewicz (2007)

In the period between 1991 and 2000, research mainly focused on the individual *Factors affecting ethical decision-making process*. In the subsequent period between 2001 and 2010, research built on the knowledge of the previous 10-year period and studied more thoroughly the individual factors, such as gender (Cohen et al., 2001; Liyanarachchi & Newdick, 2009; Marques & Azevedo-Pereira, 2009; Roxas & Stoneback, 2004) and age (Marques & Azevedo-Pereira, 2009), of which age was found to be an important determinant of relativism (Marques & Azevedo-Pereira, 2009). Regarding gender, results of some researchers show that females are more critical when evaluating ethical dilemmas and less likely to perform a questionable action described in dilemmas, when compared to males (Cohen et al., 2001). On the other hand, research by Marques and Azevedo-Pereira (2009) reveals just the opposite, in the dilemmas where gender differences exist males are more critical than females. In any case, gender results are situation-specific and have to be interpreted with caution. In addition, the question of which gender is more ethical depends on the culture (Roxas & Stoneback, 2004). Another explanation for the inconsistent results could be that most studies focus on main effects only. Liyanarachchi and Newdick (2009) show in their research that there is an interaction effect between gender and moral development on the propensity for blowing the whistle.

In addition to culture, researchers concentrated also on other situational factors, such as profession (Cohen et al., 2001), professional commitment (Elias, 2006), social pressure (O’Leary et al., 2007), and education (McManus & Subramaniam, 2009). When comparing students and professionals, the results reveal that professionals do not only rely more on utilitarianism, but are also less willing to perform a questionable action described in ethical dilemmas (Cohen et al., 2001). What is more, in Elias’ (2006) research, professionalism was found to positively influence moral judgment. The differences in moral judgment may exist due to the group situational factor, which means that individuals are more willing to take extreme actions (regardless whether ethical or unethical), while groups tend to make more neutral decisions (O’Leary et al., 2007). The research by McManus and Subramaniam (2009) confirms that ethics education at a university level can improve moral judgment. This and similar findings within the *Factors affecting ethical decision-making process* can be among the reasons why research in the period between 2001 and 2010 also developed around the topic *The need to teach ethics*.

The findings reported by the authors representing *The need to teach ethics* cluster emphasize that in order to succeed as knowledgeable professionals in a highly competitive and changeable business environment, students need to learn both technical and soft skills. Among soft skills, ethics education is crucial for the accounting profession (Dellaportas, 2006; Karaibrahimoğlu et al., 2009; McPhail, 2001; Melé, 2005; Molyneaux, 2004), hence accounting educators should strive to increase students' ethical awareness. One of the ethics education objectives, as defined by McPhail (2001), is thus the development of a broader view of the profession, in other words, students should be able to understand how their profession is positioned in a broader social and political context and develop moral sensitivity for others.

A newly developed *Perception of ethics* cluster reveals that students perceive ethics education as more important than faculty members (Adkins & Radtke, 2004) and that only 20% of accounting master’s degree students believe that ethics programs impact their decisions (Mayhew & Murphy, 2009). In any case, participation in a variety of different business courses that include ethics content may in fact lead to improved moral judgment and decreased pluralistic ignorance (Halbesleben et al., 2005).

#### Research Fields for the Period 2011–2020

To narrow down the results, based on 94 articles for the period 2011–2020, the minimum number of occurrences of keywords was defined as 5. Out of 522 keywords in this period, 23 met the threshold. For the co-word analysis, the resolution parameter was defined as 0.9 with the purpose of reducing the number of different clusters from 5 to 4 in order to achieve better interpretation.

Again, the thematic landscape presented in Fig. 7 overlaps with the clusters identified in Table 6. The co-word analysis groups the words *business ethics*, *students*, *education*, *ethics education*, *accounting ethics*, *model*, *curriculum* and *accounting* into a single theme (Fig. 7, green) that corresponds to the three clusters focusing on teaching ethics from different perspectives, namely *Accounting beyond technical skills, Integration of ethics in accounting education* and *Use of developed ethics frameworks* (Table 6). The second theme, including *decision making, business students, profession, behavior, values, sensitivity, gender, attitudes, organizations* and *ethical decision making* (Fig. 7, red), correlates with the *Factors affecting ethical decision-making process* cluster, where different factors influence the sensitivity of the ethical decision-making process. The last theme, with *perceptions, ethics, impact, professional ethics* and *accounting education* (Fig. 7, blue), corresponds to the *Perception of ethics* cluster.

Based on bibliographic coupling, the largest set of connected items consists of 93 items (Table 6) and the total number of clusters identified is seven (Fig. 8). The majority of the articles were published in the Journal of Business Ethics (23 articles), followed by the Advances in Accounting Education: Teaching and Curriculum Innovations (8 articles) and Accounting Education (7 articles).

**Table 6:** Cluster labels and articles in clusters for ethics education in accounting for the period 2011–2020

Cluster number	Color (Fig. 8)	Cluster label	Number of documents	Five most cited references
1	Orange	Accounting beyond technical skills	19	Chabrak and Craig (2013), Gordon (2011), Lehman (2013), Lehman (2014), Viviers et al. (2016)
2	Red	Factors affecting ethical decision-making process	16	Brent and Atkisson (2011); Hummel et al. (2018), Koh et al. (2011), Liu, (2018), Shawver and Miller (2017)
3	Yellow	Integration of ethics in accounting education	16	Bouten and Hoozée (2015), Kidwell et al. (2011), Loeb (2015), Sorensen et al. (2017), Tweedie et al. (2013)
4	Blue	Perception of ethics	14	Jones et al. (2014), Musbah et al. (2016), Saat et al. (2014), Tormo-Carbó et al. (2016), Waldron and Fisher (2017)
5	Purple	Use of developed ethics frameworks	13	Christensen et al. (2018), Christensen et al. (2016), Martinov-Bennie and Mladenovic (2015), Painter-Morland and Slegers (2018), Parvin et al. (2011)
6	Turquoise	Professional values	10	Albu et al. (2011), Andersen et al. (2015), Krambia-Kapardis and Zopiatis (2011), O’Leary and Stewart (2013), Sin et al. (2011)
7	Green	Lack of ethics topics	5	Al-Htaybat and Von Alberti-Alhtaybat (2015), Cameron and O’Leary, (2015), Ferguson et al. (2011), Larrán Jorge et al. (2015), Larrán et al. (2018)

Ethics education in accounting was extensively studied in the last period from 2010 to 2020, when the number of articles published quadrupled, compared to the preceding period. Similar to the previous two periods, *Factors affecting ethical decision-making process* remains to date an important subfield of research. Nevertheless, the most significant breakthrough in this period is the development of four new research subfields, namely *Accounting beyond technical skills, Integration of ethics in accounting education, Use of developed ethics frameworks* and *Professional values.* These four clusters, focusing on why and how to teach ethics, evolved from the cluster that developed during the period of corporate scandals (i.e. *The need to teach ethics)*. As ethics education in accounting is not yet at the desired level, the research in the *Lack of ethics topics* cluster, identified for the period 1991–2000*,* draws attention once again. And lastly, the *Perception of ethics* cluster grows compared to the previous period, due to the continuous changes in the environment on one hand and the constantly developing curriculums on the other.

#### Factors Affecting Ethical Decision-Making Process

Although the subfield *Factors affecting ethical decision-making process* was studied extensively in the previous two periods, the research on this topic continues, researches focus on both the individual (Nahar, 2018; Rodriguez Gomez et al., 2020) and situational factors (Driskill & Rankin, 2020; Hummel et al., 2018; Koh et al., 2011; Liu, 2018; Mladenovic et al., 2019; Nahar, 2018; Ramirez, 2017; Shawver & Miller, 2017; Taplin et al., 2018), with the latter gaining in importance. Among the situational factors, researchers have so far studied what effect do pressure (Koh et al., 2011), the importance of outcome (Koh et al., 2011), the pre-dispositional effect (Hummel et al., 2018), treatment effect (Hummel et al., 2018; Liu, 2018; Mladenovic et al., 2019; Ramirez, 2017; Shawver & Miller, 2017; Taplin et al., 2018), culture (Driskill & Rankin, 2020), and religion (Nahar, 2018) have on the ethical decision-making process. Further, building on the theories of profit maximization, Hummel et al. (2018) studied the effect of economics and business education on moral judgment. They did not find any differences in the predispositions of economics and business students, when compared to other majors and no effect of business education on moral judgment. Liu (2018) separated and studied the effects of ethics and auditing education on professional skepticism. While auditing education has no effect on professional skepticism, the effect of ethics education is, on the contrary, positive. Similarly, Mladenovic et al. (2019) confirmed that the effect of integrating ethics topic in accounting courses does positively affect the students’ ethical decision-making process. Ramirez and Palos-Sanchez (2018) went a step further and narrowed down the research focus on the effect of ethics education in higher education on willingness to comply with the law, with the results showing positive effect. If Ramirez and Palos-Sanchez (2018) defined clearly the scope of educational effect, it was Taplin et al. (2018) who narrowed down the ethics intervention to a short role-play intervention. In fact, role-playing was found to be an effective way to learn ethics. In addition, culture (Driskill & Rankin, 2020) and religion (Nahar, 2018) are reported as factors that influence the ethical decision-making process.

#### Perception of Ethics

Age and gender are reported to be important determinants of the students’ perception of the importance of accounting ethics (Tormo-Carbó et al., 2016). This perception is influenced additionally by education, meaning that it is the students who have taken an ethics course that show an interest in including ethics topics into the curricula (Tormo-Carbó et al., 2016). Focusing on the students’ perception of their peers, Costa et al. (2016) report that students believe their peers have lower ethical standards than they do. Further, students perceive serving public interest as more important than auditors do and have a greater need to establish independence rules, which can be explained by their lack of experience. In other words, while auditors become more confident in their moral judgment with experience and perceive rules as constraints on their judgment, students feel more confident by following the rules. Although the students’ commitment to the public interest and independence was higher than the auditors’, the results reveal that auditors perceive questionable practices as less ethical than students (Barrainkua & Espinosa-Pike, 2018). Similar results were found when comparing students and accountants (Waldron & Fisher, 2017).

#### Lack of Ethics Topics in the Education Process

Ample research on ethics education in accounting has already been conducted with the aim of improving it, nevertheless, the lack of ethics topics in educational process still persists. Larrán Jorge et al. (2015) researched whether accounting programs integrated ethics or corporate social responsibility as a stand-alone course. The result shows that only half of the business schools in the sample offer at least one such course. In addition, there is a negative relationship established between school size and course inclusion, with larger schools being more robust and their transformation taking more time. All in all, limited training related to corporate social responsibility is indeed observed, however, it is also evident that students are aware of its importance and have thus room to claim more ethical and social themes in the future (Larrán et al., 2018).

The generally prevailing view of accounting students is that accounting information is prepared above all for the shareholder’s needs (Ferguson et al., 2011). The author further explains that “*accounting and business education fails to address the ethical assumptions that it is underpinned by and fails to acknowledge alternative ethical frameworks*” (Ferguson et al., 2011, p. 24), which means that schools should be aware of their involvement in education of ethical accountants. All in all, the need to redesign accounting education (Al-Htaybat & Von Alberti-Alhtaybat, 2015) is evident throughout this research cluster.

#### Research Clusters Evolved from the Need to Teach Ethics

Cluster *The need to teach* ethics, identified in the preceding period of corporate scandals, developed further around four specific areas of research, namely the *Integration of ethics in accounting education, Use of developed ethics frameworks, Accounting beyond technical skills,* and *Professional values.*

The general opinion is that accounting educators not only need to move beyond teaching theory and standards to actually developing students' attitudes towards values and ethics (Caglio & Cameran, 2017), but also need to be aware that the perceived professional ethics is an important factor influencing students’ intention to major in accounting (Lee & Schmidt, 2014). Further, it is considered that while ethical values and professional identity should be developed within the university, the process itself should continue throughout the professional career, i.e. individuals need to understand their role within the wider economic and social system (Sin et al., 2011).

Technical skills are necessary, but not at all sufficient, in the accounting profession. Pierre and Rebele (2014) believe that the primary goal of accounting education should remain the development of technical skills, in the sense that students must first understand the subject/problem, before they can critically evaluate it (Pierre & Rebele, 2014). After this primary goal is achieved, educators should then start with the development of other student competencies. The problem is that through the education process students receive only limited soft skills, resulting in an expectation gap between student skills and the expectations of audit firms (Anis, 2017; Chaffer & Webb, 2017). Considering that accounting students value career growth and are willing to develop professional skills that are essential for the profession, ethical issues should certainly be integrated into accounting programs (Sarapaivanich et al., 2019). However, what raises concerns about the issue are the results of Sugahara and Boland (2011), revealing that only 55% of accounting academics agree that they should incorporate ethics topics into their curriculums.

Despite all the ample research on the topic, there is still no consensus on how ethics should be taught. Researchers recommend the use of innovative teaching methods, such as thematic approach (Blanthorne et al. 2017; Tweedie et al., 2013), active learning (Loeb, 2015), virtue ethics (Sorensen et al., 2017), giving voice to values (Christensen et al., 2018; Cote & Latham, 2016; Painter-Morland & Slegers, 2018), and role-playing (Bouten & Hoozée, 2015), rather than using the traditional ones. Another recommendation is the development of soft skills by applying real-life cases (Keevy, 2020).

Likewise, no consensus exists on whether ethics should be taught as a separate course or integrated into the curriculum (Blanthorne et al. 2017; Kelly & Earley, 2011; Needles Jr., 2014; Sugahara & Boland, 2011). Miller and Shawver (2018) studied the extent to which the Ethics Education Framework^2^ is used in curriculums and found that its use is low, but also that it is increasing. Ethics is at any rate a complex topic, as there is no unique approach to teaching it, and for this reason it is up to the educators to decide which framework is the most suitable to achieve the ethics-related goals. Moreover, as ethical frameworks influence moral judgment, ethics decision-making frameworks should not only be included in the codes of conduct of professional bodies (e.g. IFAC, APESB), but also presented to both professionals and students (Martinov-Bennie & Mladenovic, 2015).

## Discussion

Using *historiography*, we analyze the chronological development of ethics education in the field of accounting research. The theoretical background starts with Kohlberg (1969) who was the first to identify the nature of morality. Almost two decades later, Rest (1986) went further and developed a four-step model of moral development. Both Kohlberg’s moral development and the neo-Kolbergian model (based on Rest’s) theories were also outlined by DeTienne et al. (2019) as being two of the main streams of research in moral development in business ethics. The historiography analysis reveals that the other streams of the moral development research identified by DeTienne et al. (2019), including moral identity, domain theory, moral automaticity, moral schemas and moral heuristic, are not referenced in the ethics education in accounting research, which pinpoints a research focus that is narrower than the broader research field of business ethics.

**Fig. 9:**
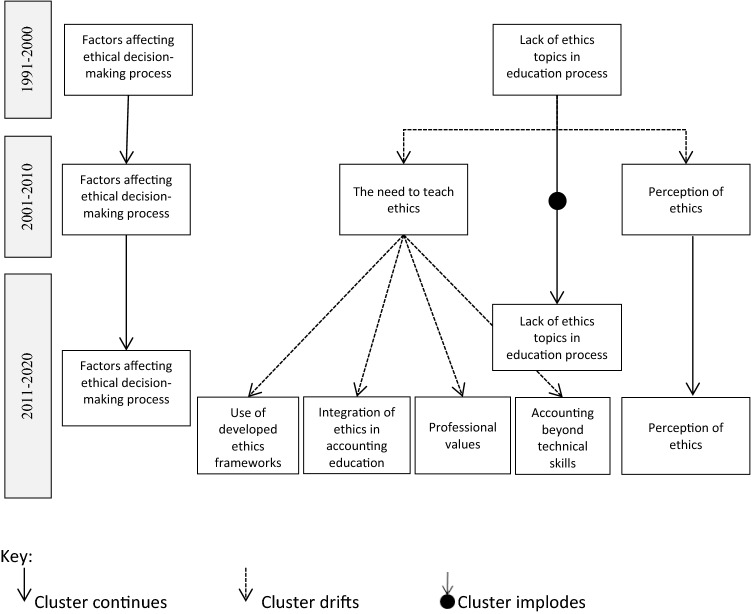
Development patterns of ethics education in accounting research

Besides research development, historiography reveals an overlap of shared knowledge between different research areas. Relatedly, the vast majority of articles (more precisely, 131 out of 133) were included in the bibliographic coupling analysis, showing bibliographic connections to other documents in the network. This positive development indicates that information and knowledge are shared across the scientific community. To see the impact of corporate scandals on research in the field, we divided the study’s timeframe into three distinct, namely the before, during, and after the major corporate scandals periods. The increase in the published articles was perhaps indeed small from the period before to the period during the corporate scandals, nevertheless, there was a major increase in the period after the scandals, when the number of the published articles quadrupled and the number of different clusters doubled, when compared to the preceding period. While some clusters persist and expand throughout the observed periods, others evolve as new research fields with a more specific focus. At any rate, the latter, despite representing a novel stream of research, build on and further develop previous knowledge. Using the bibliographic coupling analysis, we depict the development patterns of ethics education in accounting research in Fig. 9.

*Factors affecting ethical decision-making process* is identified as a continuing cluster of research. In the first period (1991–2000), this field of research prevailed with 9 primary articles (out of 15 connected items). The importance of the cluster can be outlined by the fact that all articles from the list of the 5 most cited articles in this period (Table 3) belong to this research cluster (Eynon et al., 1997; Fischer & Rosenzweig, 1995; Green & Weber, 1997; Jones & Hiltebeitel, 1995; Karcher, 1996). In the second period (2001–2010), this was still a prominent field of research with 10 primary articles (out of 23 connected items). However, only 2 articles from this cluster (Cohen et al., 2001; Roxas & Stoneback, 2004) appear among the 5 most cited articles in this period. In the last research period (2011–2020), this cluster comprises only 16 primary (out of 93 connected) articles. Another interesting observation is that while individual factors remain similarly researched between the periods, the research on the effect of situational factors, as are social pressure and treatment effect, continues to grow*.*

Interestingly, the *Lack of ethics topics in education process* cluster was noted already before the major corporate scandals. Researchers have already highlighted the importance of ethics education (Gunz & McCutcheon, 1998; Loeb, 1991; McNair & Milam, 1993), which contributed to a further development of the cluster during the period of corporate scandals (2001–2010), when the need for ethics education became thoroughly investigated. In this period, 9 primary papers (out of 23 connected items) were identified in *The need to teach ethics* cluster, three of them among the 5 most cited in the period (De Lange et al., 2006; Dellaportas, 2006; McPhail, 2001), outlining the importance of ethics education in preventing similar ethical misconduct with consequences reaching far into the future and regain public trust in the accounting profession. However, the need for ethics education also raised additional questions. How should ethics be taught? Who should teach it? Do the characteristics of the learner matter? These are some of the questions that researchers sought to answer in the last research period (2011–2020). Accordingly, *The need to teach ethics* cluster evolved into more specific research areas, including *Integration of ethics in accounting education, Use of developed ethics frameworks, Accounting beyond technical skills* and *Professional values,* comprising in total 58 primary papers (out of 93 connected items), four of them among the 5 most cited in the period (Chabrak & Craig, 2013; Martinov-Bennie & Mladenovic, 2015; O’Leary & Stewart, 2013; Tweedie et al., 2013). Among the four newly evolved clusters, *Accounting beyond technical skills* reports the highest number of primary papers (19).

All in all, the increased number of clusters, identified with the bibliographic coupling analysis, and the increased number of articles within the clusters indicate together the increasing importance of teaching ethics in accounting. Despite ample empirical evidence published in the highest quality journals, the present study reveals that the implementation of ethics topics in accounting education is, although increasing, still not at a desirable level. Based on our literature review, the contributing factors to the current state of ethics education in accounting include the following reasons: (1) some schools are slow in implementing the necessary changes due to a lack of knowledge and commitment of academics, (2) after a successful implementation of ethics into the academic syllabi, its impact on the ethics-related goals may be limited, because the selected approach disregards certain relevant (individual or situational) factors that affect the ethical decision-making process, and (3) due to the abundant empirical evidence on the topic, it may be difficult to optimize the effect of ethics education, especially when among researchers no consensus has yet been reached regarding the questions such as the development of stand-alone courses or integrating ethics topics across the curriculum, how to account for individual factors, and last but not least, what innovative teaching methods provide the best results.

The trend of the number of articles increasing in the field of ethics in accounting is also outlined in the accounting education literature review by Apostolou et al. (2010). Overall, the majority of the existing literature reviews focus on the accounting education (Apostolou et al., 2010, 2017; Rebele et al., 1991; Watson et al., 2007) or ethics in accounting (Uysal, 2010). To the best of our knowledge, the present study is novel in the sense that it combines both fields and gives a comprehensive overview of the historical development of the existing research in the field of ethics education in accounting.

## Future Research

Based on *historiography*, the research in ethics education in accounting has mostly been based on Kohlberg’s (1969) theory of moral development and Rest’s (1979) measurement instrument, DIT. Both Kohlberg’s and Rest’s theories were also outlined by DeTienne et al. (2019) as two of the main streams of the research in moral development in business ethics. However, their work covered additional theoretical foundations that are currently used in the field of business ethics, including the domain theory, moral automaticity, moral schemas, and moral heuristic. These have not yet been as well applied in the ethics education in accounting literature. Nevertheless, incorporating these novel theoretical approaches may be warranted, because they are by their nature highly multidisciplinary. DeTienne et al. (2019) outline that this area is of high interest, in addition to academics, to philosophers, psychologists, sociologists, anthropologists, neuroscientists, and other professionals. For example, approaches similar to those used in neuro-accounting apply also to moral schemas. As this stream of research focuses on the development of brain structures, it must thus be supported by both neuroscience and psychology (Narvaez, 2008). The literature review of Tank and Farrell (2021) shows that neuro-accounting has attracted the attention of researchers only from 2007, so it is not surprising that it has not yet developed in the subfield of ethics education. Future research could therefore expand the existing theoretical background so as to include other theories that are not yet as well applied, which could lead to additional clusters within the historiography of ethics education in accounting.

Based on bibliographic coupling that presents the development patterns in the field of research, to encourage future research we next develop a research agenda. The past expansion and the current developments within the *Factors affecting ethical decision-making process* cluster suggest that this cluster is to persist in the future. We can nonetheless still expect novel research focusing on new factors that were disregarded or insufficiently investigated in the previous decades. In recent years, country-specific issues have attracted the interest of researchers (Arfaoui et al., 2016; Driskill & Rankin, 2020; Marzuki et al., 2017; Mohd Ghazali, 2015), however, the consistency of the results and their implications are still lacking. Moreover, an increase in the research related to the Islamic religion, conducted over the past decade (Musbah et al., 2016; Nahar, 2018; Zubairu, 2016), may indicate that the effect of different religions on the ethical decision-making process could become a prominent area of future research within this cluster.

In the last period (2011–2020), *Use of developed ethics frameworks* and *Integration of ethics in accounting education* were among the clusters that developed from *The need to teach ethics* cluster, which evolved during the second period (2001–2010). The common characteristic of the two clusters is their focus on the course design. It is therefore plausible to expect that the clusters will merge to form a prominent *How to teach ethics* cluster, thus reaching beyond the research questions of the two previous clusters by incorporating current developments, also related to the outbreak of Covid-19, into teaching. What could become a prominent research area within the new cluster is the effect of the online ethics courses. Due to the Covid-19 outbreak in March 2020, education worldwide was forced to go online in the spring of the same year (Alassaf & Szalay, 2020; Sun et al., 2020). While the vast majority of the existing research has so far focused on the in-class ethics education (Arfaoui et al., 2016; Shawver & Miller, 2017), we expect a growing body of literature to focus mainly on the online methods. In fact, recent research (Sorensen et al., 2017) has already denoted this trend. Research questions in these clusters have so far included the identification of, on one hand, the most effective teaching approaches that are applied in ethics education, such as the thematic approach (Tweedie et al., 2013), active learning (Loeb, 2015), role-playing (Bouten & Hoozée, 2015), and on the other hand, the research questions related to whether ethics should be taught as a stand-alone course or using an integrated approach. This field merits additional insight, as despite extensive research no consensus on the topic has been reached to date (Dellaportas, 2006; Martinov-Bennie & Mladenovic, 2015). In addition, while most researchers have focused on comparing the effectiveness of traditional and innovative teaching methods, there is a lack of comparison between innovative methods.

The specific part of the *Integration of ethics in accounting education* cluster, which relates to virtue ethics (Sorensen et al., 2017), could merge with the *Professional values* cluster to form a new *Professional values and virtues* cluster*.* Moral virtues focus on character development and represent a permanent attitude towards moral behavior. Since the objectives of ethics education are to increase moral sensitivity, help individuals to make moral judgments, improve moral behavior and stimulate moral virtues, moral values and virtues are closely related and should therefore be treated in ethics education (Melé, 2005). The importance of moral virtues has been frequently addressed in the field of medical ethics (Toon, 2014), however, the existing lack of comparable research in ethics education in accounting can be identified as another research gap.

In the last period (2011–2020), the importance of developing soft skills in accounting education was addressed in the *Accounting beyond technical skills* cluster. This stream of research originated in the gap observed between the skills of accounting students and expectations of employers. Ma (2009), who examined the status of the business ethics research, reported that in the all-encompassing pursuit of profits in capitalist economies, the effect of business ethics on financial performance became one of the main determinants of the promotion of ethical behavior. Similarly, a new stream of ethics education in the accounting research, included in the *Practical importance of ethics in accounting* cluster, could investigate whether (and to what extent) the motivation for promoting soft skills, together with ethics and moral skills, has been redefined to include its effects on corporate financial performance.

**Fig. 10:**
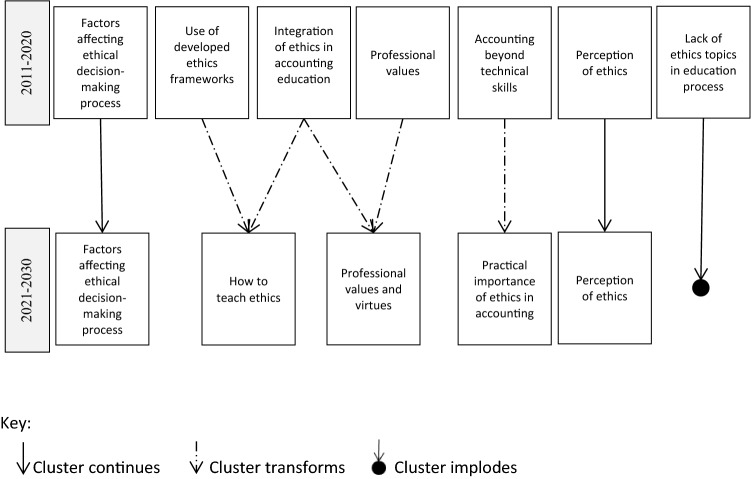
Future research development patterns of ethics education in accounting research

While the *Lack of ethics topics in education process* cluster seems to evolve and implode in cycles, based on the latest research findings on the existent situation in accounting education, *Perception of ethics* is, on the contrary, a continuous cluster that not only provides an overview of the current state of ethics and its improvement, but is also expected to continue to exist in the future, due to continuous changes in the environment and the constantly developing curriculums. Future research development patterns of ethics education in the accounting research are presented in Fig. 10.

## Conclusion and Limitations

Looking at the existing research in the field of ethics education in accounting reveals a vast amount of work that researchers have already done on the topic. The objective of this paper however is to trace the evolution of ethics education in accounting, define the thematic landscapes and outline the subfields that constitute the ethics education in the accounting research. We attempted to accomplish this objective by performing historiography, bibliographic coupling and co-word analysis. Dividing the study’s timeframe into three different, i.e. the before, during and after the major corporate accounting scandals, periods allowed us to see the true impact of the corporate accounting scandals on the investigated research area. Moreover, our review highlights the most influential articles and journals in the field.

The theoretical backgrounds used in ethics education in accounting research are based on the fundamental theories from the field of business ethics, while research does not yet build on the newly developed concepts, such as moral identity, domain theory, moral automaticity, moral schemas and moral heuristics (DeTienne et al., 2019). These interdisciplinary approaches that have been applied in the business ethics research provide scholars with a venue of further research.

The present study gives a comprehensive overview of the topic and thus contributes to a more effective and efficient implementation of ethics education in accounting and future research. Although the importance of ethics education was outlined even before the corporate accounting scandals, the number of published articles quadrupled while the number of different clusters doubled in the period in the period following the scandals in comparison to the previous period, the implementation of ethics topics in accounting education is not yet at a desired level, due to a lack of knowledge in its implementation on one side and a lack of commitment from academics on the other. Moreover, the true impact of the implemented ethics education is still limited as a consequence of the numerous factors affecting its success. In each period, researchers describe additional factors that affect the ethical decision-making process, among which situational factors gain in their importance. The period of the corporate accounting scandals outlined *The need to teach ethics*, which resulted in four research areas developed in the last period, namely *Integration of ethics in accounting education*, *Use of developed ethics frameworks*, *Accounting beyond technical skills*, and *Professional values.* To improve the effectiveness of ethics education, educators should pay special attention to the course design and its development, especially in terms of the content and structure of the course, the ethics frameworks use and the teaching methods, with researchers recommending the use of innovative rather than traditional methods.

We expect researchers to continue studying the individual and situational factors, with the emphasis on the latter. Further, innovative teaching methods are proving to be more effective than the traditional ones, however, there is a lack of comparison between innovative methods. Moreover, the vast majority of research to date has focused on teaching ethics in the classroom. Due to the Covid-19 outbreak in March of 2020, which forced education to go online, we assume a growing body of literature to focus on online methods.

Like many before, this study too faces several limitations. Firstly, not all the existing articles related to the ethics education in accounting are necessarily included in the research. We decided to use as the database source the articles published in the English language in the Web of Science, within the science categories that comprise business and finance, business, education educational research and ethics. The final sample thus consist of the articles published between 1991 and 2020, and it is a fact that using different keywords or methods, or different research periods or science categories might result in the discovery of connections and developments invisible to this study. Secondly, using bibliographic coupling as a method for our research has proven to have its own limitations, since the analysis treats all citations equally and does not distinguish between different reasons for citing (support vs. criticism).

## Footnotes

1 Using asterisk in the search engine enabled the inclusion of related words (e.g. ethics, ethical, ethicality).

2 The framework was developed by Cooper et al. (2008) and expanded by Dellaportas et al. (2011). It is based on Rest’s (1986) four-component model of ethical decision making. The framework offers the students and professional accountants a learning tool to identify, analyze and resolve ethical dilemmas (Dellaportas et al., 2011).
